# Association of metabolic syndrome severity with cognitive decline among Chinese older adults: evidence from two prospective cohort studies

**DOI:** 10.3389/fnins.2026.1780804

**Published:** 2026-03-17

**Authors:** Ming Chen, Lu Liu, Na Liu, Ji-Wen Che, Yuan-Yuan Peng, Yan Zeng

**Affiliations:** 1 Hubei Provincial Clinical Research Center for Alzheimer’s Disease, Tianyou Hospital, School of Medicine, Wuhan University of Science and Technology, Wuhan, China; 2 Brain Science and Advanced Technology Institute, Wuhan University of Science and Technology, Wuhan, China

**Keywords:** CHARLS, cognitive decline, dual-cohort study, longitudinal study, metabolic syndrome severity

## Abstract

**Introduction:**

The relationship between metabolic syndrome (MetS) severity and cognitive decline is not well understood, largely because the traditional binary diagnosis of MetS is unable to capture its continuous nature.

**Methods:**

This longitudinal study included 1,191 participants aged ≥ 60 years from the Hubei Memory and Aging Cohort Study (HMACS, 2016–2024) and 2,281 participants from the China Health and Retirement Longitudinal Study (CHARLS, 2011–2020). Annual rates of cognitive decline were calculated using standardized z-scores. An age-sex-ethnicity-specific MetS score model was used to calculate the MetS score for assessing MetS severity. The Cumulative MetS score was defined as (baseline MetS score + final MetS score)/2 × follow-up duration. The association between baseline and cumulative MetS score and annual rates of cognitive decline was evaluated using linear mixed models. Results from the two cohorts were pooled through random-effects meta-analysis. Sensitivity analyses included re-clustering of MetS trajectories and exclusion of participants with younger than 65 years and baseline cognitive impairment.

**Results:**

A higher baseline MetS score was associated with a faster rate of global cognitive decline [*β_*HMACS*_* = –0.052 (–0.073, –0.031); *β_*CHARLS*_* = –0.028 (–0.041, –0.015); pooled β = –0.038 (–0.062, –0.015) z-score/year]. Participants in the highest quartile (Q4) of cumulative MetS score showed a more rapid annual decline in memory compared with those in the lowest quartile (Q1) [*β_*HMACS*_* = –0.100 (–0.16, –0.041); *β_*CHARLS*_* = –0.118 (–0.149, –0.086); pooled β = –0.114 (–0.142, –0.086) z-score/year]. These associations remained stable across all sensitivity analyses.

**Discussion:**

Greater MetS severity is linked to a faster rate of cognitive decline in Chinese older adults, underscoring the urgent need for early detection of MetS and targeted interventions to mitigate the risk of cognitive deterioration.

## 1 Introduction

The rapid aging of the global population is turning cognitive decline and dementia into increasingly public health concerns that pose major challenges to aging societies (Ding et al., 2022; Livingston et al., 2020; Sexton et al., 2022). It is estimated that in 2019, among the 57.4 million people living with dementia worldwide, more than one-quarter (13.1 million) were in China (Li X. et al., 2022), and this burden is expected to continue rising (Huang Y. et al., 2022). In the absence of effective curative treatments, early identification and intervention of potential risk factors are essential to delay cognitive decline and reduce the incidence of dementia (Alzheimers Dementia, 2024).

Metabolic syndrome (MetS), another major public health concern, affecting approximately 25% of adults worldwide (Qureshi et al., 2024a) is diagnosed when at least three of the following conditions are present: increased waist circumference, elevated triglycerides, high blood pressure, high blood glucose, and low high-density lipoprotein cholesterol (HDL-C) levels (Alberti et al., 2009). It is a well-established modifiable risk factor for cardiovascular and cerebrovascular diseases, making it a critical target for preventive strategies (Rodriguez-Colon et al., 2009). In recent years, increasing evidence has shown that MetS is not only strongly associated with cardiovascular disease but may also adversely affect cognitive function, increasing the risk of cognitive decline and dementia (Lee et al., 2020; Pillai et al., 2023; Yoo et al., 2022; Zuo et al., 2024). Recent large-scale multicenter cohort studies have further confirmed a significant association between cardiovascular-kidney-metabolic syndrome and cognitive decline or incident dementia (Xie et al., 2025). Mechanistic studies suggest that MetS and its components exert both additive/synergistic effects on brain microstructure, thereby accelerating brain aging and cognitive decline (Alfaro et al., 2018), with white matter hyperintensities serving as a mediating variable between MetS and cognitive decline (Zheng et al., 2023). However, a systematic review has summarized that current data regarding the impact of MetS on cognition in older adults remains inconclusive and is based on conflicting evidence. Differential effects of individual MetS components and age-related factors in study samples may account for the divergent findings across studies (Assuncao et al., 2018). In particular, among older adults in China, some studies have not found significant associations between MetS or overall cardiometabolic risk and cognitive decline (Chen et al., 2016; Srikantha et al., 2024; Wu Q. et al., 2024). Notably, these studies were mostly cross-sectional in design and defined exposures using binary classifications. This highlights the need for more refined measures of metabolic risk and longitudinal study designs to systematically examine the relationship between MetS and changes in cognitive function over time.

Furthermore, an important limitation of existing research is that most studies have relied on the binary diagnosis of MetS or its individual components (Machado-Fragua et al., 2022; Qureshi et al., 2024a; Qureshi et al., 2024b; Zuin et al., 2021). Evidence suggests that even during the preclinical stage of MetS, exposure to a single metabolic abnormality is associated with a significantly increased risk of developing dementia and Alzheimer’s disease (AD) later in life (Cho et al., 2021). These findings suggest that metabolic disturbances occurring in the early stages of MetS may have a significant impact on future cognitive decline. However, the binary classification of MetS does not capture the continuum of metabolic severity nor does it account for variations across ethnicity, sex, and age, thus providing an incomplete perspective on the nuanced progression of the syndrome. To address these limitations, Yang et al. developed an age-sex-ethnicity-specific MetS score for the Chinese population to directly quantify subtle, dynamic changes in MetS progression (Yang et al., 2023), enabling a dynamic assessment of subtle changes in MetS progression. However, no large-scale population studies have yet evaluated the association between dynamic changes in MetS progression and the annual rate of cognitive decline.

This longitudinal study respectively based on a recently established cognitive cohort in central-western China, the Hubei Memory and Aging Cohort Study (HMACS, 2016–2024), and a nationally representative Chinese cohort, the China Health and Retirement Longitudinal Study (CHARLS, 2011–2020), comprehensively examines the relationship between age-sex-ethnicity-specific MetS score and annual rates of cognitive decline, while also considering the impact of cumulative burden of MetS on cognitive decline. Results from the two cohorts were combined through random-effects meta-analysis. We hypothesized that a higher MetS score would be associated with faster cognitive decline.

## 2 Materials and methods

### 2.1 Study population

Participants were derived from two prospective cohort studies: the HMACS and the CHARLS. The HMACS (Chinese Clinical Trial Registry ID: ChiCTR180001916)^1^ is the first large-scale, community-based prospective memory and aging cohort in central China. Launched in 2016, the study used cluster sampling to enroll participants aged 65 years and older from 60 administrative villages in Dawu County, Hubei Province, and 34 street communities in Wuhan City. It also included chronic disease patients aged 55 and above with diabetes, hypertension, or stroke. Recruitment and baseline data collection were completed in 2018, and the detailed study protocol has been described in previous publications (Cheng et al., 2023; Li L. et al., 2022; Zhang et al., 2025). CHARLS is a nationally representative longitudinal survey targeting adults aged 45 years and older and their spouses across China (Zhao et al., 2014). It includes data from Wave 1 (2011–2012), Wave 2 (2013–2014), Wave 3 (2015–2016), Wave 4 (2018) and Wave 5 (2020). Both cohorts collected sociodemographic, lifestyle, and health-related information through face-to-face interviews. Detailed descriptions of the cohorts have been provided elsewhere (Li L. et al., 2022; Zhao et al., 2014). The HMACS protocol was approved by the Ethics Committee of Wuhan University of Science and Technology (approval number 201845), and CHARLS was approved by the Institutional Review Board of Peking University (approval number IRB00001052–11015). Written informed consent was obtained from all participants.

In this study, to maximize the follow-up population in HMACS, we used participants who were observed for the first time as the baseline population and followed them through Wave 8 (2024). In CHARLS, participants from the Wave 1 (2011–2012) were used as the baseline population and followed through Wave 5 (2020). Further details on the study design, including inclusion and exclusion criteria, are provided in Supplementary Figure 1. Participants were excluded if they were younger than 60 years, had missing baseline MetS or cognitive data, had pre-existing memory-related diseases, had fewer than two cognitive assessments during follow-up, or were lost to follow-up. As a result, participants with complete baseline MetS score data were included in the first-stage analysis (*n* = 1,191 in HMACS; *n* = 2,281 in CHARLS). Among them, those with available follow-up MetS score data were included in the second-stage analysis (*n* = 1,185 in HMACS; *n* = 1,408 in CHARLS).

### 2.2 Cognitive evaluation

The outcome of this study was the annual rate of cognitive decline, estimated using repeated measures of cognitive function from the HMACS and the CHARLS, which included two domains: memory and executive function. To reflect the annual rate of cognitive decline and allow comparability between HMACS and CHARLS, standardized cognitive Z-scores were calculated. Z-scores for memory and executive function were derived using the mean and standard deviation (SD) of the baseline raw cognitive scores in each cohort (Li et al., 2025). In HMACS, episodic memory was assessed using the Auditory Verbal Learning Test (AVLT), which involved three immediate recall trials of 12 words from three semantic categories, followed by delayed recall trials at 5 and 20 min. Since the raw scores of immediate recall may be relatively high, immediate (0–36) and delayed (0–24) recall were standardized separately to prevent immediate recall from having excessive weight in the total score, and the mean of these standardized scores was used as the final memory Z-score (Ma et al., 2015; Zhang et al., 2023). Executive function was assessed using standardized scores from the Trail Making Test (TMT) Parts A and B tests. In CHARLS, episodic memory was assessed through immediate and delayed recall. Ten unrelated Chinese words were read to each participant, and the memory ability was evaluated by counting how many words could be recalled immediately (immediate word recall) and 4 min later (delayed word recall). Scores for immediate recall and delayed recall were averaged to obtain a single memory score (0–10), which was then converted into a Z-score (Ding et al., 2023). To improve comparability between the 2018 and 2020 assessments, an equipercentile equating algorithm was applied (Wu Y. et al., 2024). Executive function was evaluated using three tasks: time orientation (0–5), numerical ability (0–5), and the pentagon drawing test (0–1), yielding a total score range of 0–11. A Z-score was then calculated based on the total score to assess executive function. Global cognitive function was defined as the mean of the Z-scores for memory and executive function and then re-standardizing this using baseline values of the global cognitive z-scores (Ding et al., 2023). Higher Z-scores for memory, executive, and global cognitive scores indicated better performance in each respective domain.

### 2.3 Assessment of MetS score and the cumulative MetS score

We calculated the MetS score based on the age-sex-ethnicity-specific MetS score model developed by Yang et al. for Chinese adults (Yang et al., 2023). The MetS score was derived as a weighted sum of waist circumference (WC), triglycerides (TG), HDL-C, mean arterial pressure (MAP), and fasting blood glucose (FBG). In this model, separate regression equations were established according to age ( < 60 years and ≥ 60 years), sex (male and female), and ethnicity (Han and minority). Because all participants in the present study were aged ≥ 60 years, we applied the equations corresponding to the ≥ 60-year age group. Following the original modeling strategy, sex-ethnicity-specific equations were used according to each participant’s sex (male or female) and ethnicity (Han or minority). Detailed methods are provided in Supplementary Table 5. To reduce potential bias arising from heterogeneous health conditions (e.g., undernutrition or frailty) that may be represented at the lower end of the MetS score distribution, we standardized the continuous MetS score using the baseline mean and SD. We evaluated the cumulative burden of MetS over follow-up by calculating the Cumulative MetS score (Cui et al., 2022; Huo et al., 2023; Zeng et al., 2024): (baseline MetS score + final MetS score)/2 × follow-up duration.

### 2.4 Covariates

The following baseline characteristics were identified as covariates, mainly covering sociodemographic factors, lifestyle behaviors, and health status. Sociodemographic factors included age, sex, residence (urban vs. rural), marital status (married or partnered vs. others), educational level (categorized as elementary, middle, high school, or college and above), and income (low, middle, and high income levels). Lifestyle factors included smoking status (never vs. former or current), alcohol consumption (never vs. former or current), and engagement in moderate physical activity at least weekly (yes/no). Health status included self-reported heart-related diseases (yes vs. no) and stroke (yes vs. no).

### 2.5 Statistical analysis

In the descriptive analysis, continuous variables were presented as means [standard deviations (SDs)], and categorical variables were presented as counts (percentages). Baseline characteristics across groups were compared using one-way ANOVA for continuous variables and chi-square tests for categorical variables. Continuous MetS scores were standardized using z-scores based on the baseline mean and SD for further analyses.

A linear mixed-effects model was used to examine the association between MetS score and annual cognitive decline, accounting for individual variability as random effects. Specifically, random intercepts and slopes were included to capture individual differences at baseline and over time. An unstructured covariance matrix was assumed to model the covariance between random effects, allowing flexible representation of within-person correlations in repeated measures. All available repeated measurements of cognitive function (including baseline values) were included as the outcome. MetS score, time (follow-up duration), their interaction term (MetS score × time), and covariates were entered as fixed effects. Here, MetS score referred to the baseline measurement, and the regression coefficient of the interaction term represented the longitudinal association between MetS score and the annual rate of cognitive decline, with positive coefficients indicating a slower decline and negative coefficients indicating a faster decline. The same analytical approach was applied to Cumulative MetS score. Results from the two cohorts were then pooled using an inverse-variance weighted random-effects model, accounting for between-study heterogeneity (Huang et al., 2024). Using the same modeling strategy, additional analyses were performed with memory function and executive function as outcome variables to explore their longitudinal associations with MetS score and Cumulative MetS score.

In the stratified interaction analysis, the following baseline characteristics were considered as potential effect modifiers: age ( < 75 vs. ≥ 75 years), sex (male vs. female), residence (urban vs. rural), marital status (married or with partner vs. other), educational level ( ≤ primary school, middle school, high school, ≥ college), income level (low, middle and high levels), smoking status (never vs. former or current), alcohol consumption (never vs. former or current), weekly engagement in at least one session of moderate physical activity (yes vs. no), and self-reported history of heart-related disease and stroke (yes vs. no). For each covariate, an interaction term between baseline MetS score and the covariate (MetS score × covariate) was constructed and included in a linear mixed-effects model to evaluate potential subgroup differences. Fixed effects in the model included baseline MetS score, follow-up time, other covariates except the current interaction variable, and the interaction term. Random effects included individual random intercepts and slopes, and an unstructured covariance matrix was used to account for within-person correlations in repeated measurements. The statistical significance of the interaction term was assessed using Type III Wald tests (P for interaction). To visually present the longitudinal effects in each subgroup, the main analysis model was fitted separately within each category of the stratification variables to estimate the effect of MetS score on the rate of cognitive decline in different subgroups. Additionally, in the CHARLS cohort, to explore whether specific metabolic abnormalities amplified the effect of cumulative metabolic burden, three-way interaction terms (MetS score × time × metabolic component) were constructed for five metabolic components defined according to the 2024 criteria of the Chinese Diabetes Society (CDS) for metabolic syndrome (Chinese Diabetes Society, 2025). These components included waist circumference, elevated triglycerides, high blood pressure, high blood glucose, and low HDL-C levels. Each interaction term was incorporated into separate linear mixed-effects models to evaluate potential subgroup differences in the longitudinal association between MetS score and cognitive decline over time.

For sensitivity analyses, the main analyses were repeated as follows: first, we applied an unsupervised machine learning approach, K-means clustering using Euclidean distance, to classify the change trajectory in MetS score (MetS score trajectory) and assess its trajectory patterns (Zeng et al., 2024). The K-means algorithm partitions the dataset into K clusters by minimizing the within-cluster sum of squares. The optimal number of clusters (*K* = 4) was determined using the elbow method; second, given that the HMACS cohort included community-dwelling older adults aged ≥ 65 years as well as individuals aged 55–64 years with chronic diseases, the inclusion of participants aged 60–64 years in our primary analysis may have introduced selection bias, as individuals in this age group were more likely to have pre-existing chronic conditions. To minimize this potential bias and improve comparability between the two cohorts, we conducted a sensitivity analysis restricted to participants aged ≥ 65 years in both HMACS and CHARLS. In addition, based on previous studies, cognitive impairment was defined as a global cognitive Z-score at least 1.5 SDs below the population mean (Li C. et al., 2022; Li et al., 2025), and the main analyses were repeated after excluding participants with cognitive impairment. Ultimately, participants aged ≥ 65 years and free of cognitive impairment at baseline with complete baseline MetS score were included in the first-stage analysis (*n* = 1,102 in HMACS; *n* = 1,055 in CHARLS). Among them, those with available follow-up MetS score data were included in the second-stage analysis (*n* = 1,097 in HMACS; *n* = 629 in CHARLS). All statistical analyses were performed using R software (version 4.4.3), and two-sided *P* < 0.05 were considered statistically significant.

## 3 Results

### 3.1 Baseline characteristics

A total of 1,191 participants (72.0 ± 5.2 years, 58.9% female) from the HMACS, all of whom were of Han ethnicity, and 2,281 participants (66.0 ± 5.0 years, 54.4% female) from the CHARLS, of whom 94.3% were of Han ethnicity, were included in the final analysis. Table 1 presents the baseline characteristics of participants according to quartiles of baseline MetS score. In the HMACS cohort, participants in the highest MetS score quartile (Q4) were younger, more likely to reside in urban areas, and had higher proportions of being married or partnered, had higher income levels, and having a history of stroke compared with those in the lowest quartile (Q1). Similarly, in the CHARLS cohort, participants in Q4 were also younger and had higher income levels and a higher prevalence of stroke. In both the HMACS and CHARLS cohorts, the proportion of female participants was lower in the highest quartile of MetS score (Q4) compared with the lowest quartile (Q1). However, compared with the HMACS cohort, participants in the CHARLS Q4 group included a higher proportion of rural residents, and a greater proportion of individuals who were physically inactive, non-drinkers, or non-smokers, along with a higher prevalence of heart-related diseases. Baseline characteristics for the transformation analyses are provided in Supplementary Tables 1–3.

**TABLE 1:** Baseline characteristics of the study population by quartiles of baseline MetS score in HMACS (*N* = 1,191) and CHARLS (*N* = 2,281).

Characteristic	Quartiles of baseline MetS score					*p*-value
	Overall	Q1	Q2	Q3	Q4	
Hubei Memory and Aging Cohort Study (HMACS)						
N	1191	298	298	298	297	
Age, mean (SD), year	72.0 (5.2)	72.2 (5.3)	72.4 (5.1)	71.8 (5.3)	71.4 (5.0)	0.072
Age, n (%)						0.007
< 75	839 (70.4)	204 (68.5)	191 (64.1)	217 (72.8)	227 (76.4)	
≥ 75	352 (29.6)	94 (31.5)	107 (35.9)	81 (27.2)	70 (23.6)	
Female, n (%)	702 (58.9)	180 (60.4)	177 (59.4)	170 (57.0)	175 (58.9)	0.866
Residence, n (%)						0.009
Urban	701 (58.9)	159 (53.4)	165 (55.4)	182 (61.1)	195 (65.7)	
Rural	490 (41.1)	139 (46.6)	133 (44.6)	116 (38.9)	102 (34.3)	
Educational level, n (%)						0.341
Elementary school or below	508 (42.7)	138 (46.3)	138 (46.3)	118 (39.6)	114 (38.4)	
Middle school	269 (22.6)	66 (22.1)	65 (21.8)	73 (24.5)	65 (21.9)	
High school	260 (21.8)	62 (20.8)	54 (18.1)	69 (23.2)	75 (25.3)	
≥ College	154 (12.9)	32 (10.7)	41 (13.8)	38 (12.8)	43 (14.5)	
Marital status, n (%)						0.011
Married or partnered	865 (73.5)	195 (66.1)	224 (76.2)	226 (76.4)	220 (75.3)	
Other	312 (26.5)	100 (33.9)	70 (23.8)	70 (23.6)	72 (24.7)	
Physically active, n (%)	853 (72.3)	207 (69.9)	209 (71.1)	214 (72.1)	223 (76.1)	0.365
Alcohol consumption, n (%)	326 (27.7)	95 (32.2)	81 (27.6)	71 (24.0)	79 (27.0)	0.164
Current smoking, n (%)	305 (25.8)	77 (26.0)	81 (27.6)	73 (24.6)	74 (25.2)	0.857
Income, n (%)						0.011
Low	397 (34.9)	122 (43.0)	102 (36.3)	86 (30.2)	87 (30.4)	
Middle	455 (40.1)	105 (37.0)	116 (41.3)	119 (41.8)	115 (40.2)	
High	284 (25.0)	57 (20.1)	63 (22.4)	80 (28.1)	84 (29.4)	
Heart-related diseases, n (%)	227 (19.4)	53 (18.0)	51 (17.5)	65 (22.0)	58 (20.2)	0.485
Stroke, n (%)	214 (18.4)	38 (12.9)	64 (22.0)	59 (20.0)	53 (18.5)	0.031
Han ethnicity, n(%)	1191 (100)	298 (100)	298 (100)	298 (100)	297 (100)	
MetS score, mean (SD)	0.199 (0.864)	–0.753 (0.539)	–0.055 (0.112)	0.368 (0.136)	1.238 (0.766)	
China Health and Retirement Longitudinal Study (CHARLS)						
N	2,281	559	583	564	575	0.019
Age, mean (SD), year	66.0 (5.0)	66.3 (5.1)	65.8 (4.8)	66.3 (5.4)	65.5 (4.9)	
Age, n (%)						0.430
< 75	2,119 (92.9)	516 (92.3)	549 (94.2)	518 (91.8)	536 (93.2)	
≥ 75	162 (7.1)	43 (7.7)	34 (5.8)	46 (8.2)	39 (6.8)	
Female, n (%)	1241 (54.4)	354 (63.3)	339 (58.1)	286 (50.7)	262 (45.6)	< 0.001
Residence, n (%)						< 0.001
Urban	1,456 (63.8)	401 (71.7)	398 (68.3)	345 (61.2)	312 (54.3)	
Rural	825 (36.2)	158 (28.3)	185 (31.7)	219 (38.8)	263 (45.7)	
Educational level, n (%)						0.744
Elementary school or below	1,091 (47.8)	279 (49.9)	261 (44.8)	278 (49.3)	273 (47.5)	
Middle school	695 (30.5)	171 (30.6)	192 (32.9)	160 (28.4)	172 (29.9)	
High school	351 (15.4)	77 (13.8)	94 (16.1)	90 (16.0)	90 (15.7)	
≥ College	144 (6.3)	32 (5.7)	36 (6.2)	36 (6.4)	40 (7.0)	
Marital status, n (%)						0.494
Married or partnered	1,960 (85.9)	483 (86.4)	509 (87.3)	475 (84.2)	493 (85.7)	
Other	321 (14.1)	76 (13.6)	74 (12.7)	89 (15.8)	82 (14.3)	
Physically active, n (%)	1,975 (86.7)	503 (90.0)	507 (87.0)	483 (85.8)	482 (84.0)	0.025
Alcohol consumption, n (%)	1004 (44.0)	301 (53.8)	248 (42.5)	234 (41.6)	221 (38.4)	< 0.001
Current smoking, n (%)	1,022 (44.8)	288 (51.5)	271 (46.5)	238 (42.2)	225 (39.1)	< 0.001
Income, n (%)						0.034
Low	671 (32.4)	163 (32.4)	191 (35.8)	168 (32.5)	149 (28.8)	
Middle	735 (35.5)	200 (39.8)	178 (33.3)	176 (34.0)	181 (35.0)	
High	665 (32.1)	140 (27.8)	165 (30.9)	173 (33.5)	187 (36.2)	
Heart-related diseases, n (%)	330 (14.5)	61 (10.9)	81 (14.0)	78 (13.9)	110 (19.2)	0.001
Stroke, n (%)	61 (2.7)	11 (2.0)	16 (2.7)	16 (2.8)	18 (3.1)	0.657
Han ethnicity, n(%)	2151 (94.3)	522 (93.4)	552 (94.7)	536 (95.0)	541 (94.1)	0.647
MetS score, mean (SD)	0.316 (0.985)	–0.814 (0.387)	–0.024 (0.174)	0.521 (0.169)	1.560 (0.836)	

Categorical variables were presented as numbers (percentage, %), and continuous variables were presented as mean (standard deviation, SD). *P* values for differences between groups were derived using Pearson’s Chi-squared test for categorical variables and the Kruskal–Wallis rank sum test for continuous variables. This Other marital status refers to divorced, separated, widowed, or never married statuses. *MetS score*, metabolic syndrome score; *SD*, standard deviation.

### 3.2 Association between baseline MetS score and cognitive decline

Table 2 presents the associations between baseline MetS score and cognitive function as estimated by the linear mixed-effects models. After adjusting for all covariates, each 1-SD increase in MetS score was associated with a faster decline in cognitive function [*β*_HMACS_ = –0.052 (–0.073, –0.031); *β*_CHARLS_ = –0.028 (–0.041, –0.015); pooled *β* = –0.038 (–0.062, –0.015) z-score/year]. When the continuous MetS score was categorized into quartiles, participants in the highest quartile (Q4) showed a significantly faster decline in global cognition compared with those in the lowest quartile (Q1) [*β*_HMACS_ = –0.118 (–0.173, –0.063); *β*_CHARLS_ = –0.112 (–0.129, –0.096); pooled *β* = –0.113 (–0.129, –0.096) z-score/year]. The linear trend was statistically significant in both cohorts and in the pooled analysis (*P*_HMACS_ < 0.001, *P*_CHARLS_ < 0.001, pooled *P* < 0.001). Similar results were observed for memory function, as shown in Table 3. After full adjustment, each 1-SD increase in MetS score was associated with a faster decline in memory performance [*β*_HMACS_ = –0.049 (–0.075, –0.024); *β*_CHARLS_ = –0.037 (-0.052, –0.022); pooled *β* = –0.040 (–0.054, –0.027) z-score/year]. Participants in Q4 also showed a significantly faster decline in memory function compared with those in Q1 [*β*_HMACS_ = –0.114 (–0.181, –0.046); *β*_CHARLS_ = –0.132 (–0.156, –0.108); pooled *β* = –0.130 (–0.152, –0.108) z-score/year], with significant linear trends in both cohorts and the pooled analysis (all *P* < 0.001). For executive function, the association with continuous MetS score did not reach statistical significance (Table 4). However, when analyzed by quartiles, participants in Q4 experienced a significantly faster decline than those in Q1 [*β*_HMACS_ = –0.114 (–0.187, –0.041); *β*_CHARLS_ = –0.049 (–0.062, –0.037), pooled *β* = –0.071 (–0.132, –0.011) z-score/year]. The linear trend remained statistically significant in both cohorts (*P*_HMACS_ < 0.001, *P*_CHARLS_ < 0.001, pooled *P* = 0.017).

**TABLE 2:** Longitudinal association between different classes of MetS score and rate of change in global cognitive function.

Characteristic	HMACS		CHARLS		Pooled results	
	β (95% CI)	*p*-value	β (95% CI)	*p*-value	β (95% CI)	*p*-value
MetS score, per SD	–0.052 (–0.073, –0.031)	< 0.001	–0.028 (–0.041, –0.015)	< 0.001	–0.038 (–0.062, –0.015)	0.001
MetS score, quartiles						
Q1	Ref.	Ref.	Ref.	Ref.	Ref.	Ref.
Q2	0.001 (–0.054, 0.056)	0.972	–0.025 (–0.041, –0.008)	0.003	–0.023 (–0.038, –0.008)	0.003
Q3	–0.107 (–0.16, –0.054)	< 0.001	–0.147 (–0.165, –0.130)	< 0.001	–0.135 (–0.171, –0.099)	< 0.001
Q4	–0.118 (–0.173, –0.063)	< 0.001	–0.112 (–0.129, –0.096)	< 0.001	–0.113 (–0.129, –0.096)	< 0.001
*P* for trend		< 0.001		<0.001		< 0.001
Cumulative MetS score, per SD	–0.02 (–0.029, –0.011)	< 0.001	–0.010 (–0.013, –0.007)	< 0.001	–0.014 (–0.023, –0.004)	0.004
Cumulative MetS score, quartiles						
Q1	Ref.	Ref.	Ref.	Ref.	Ref.	Ref.
Q2	–0.027 (–0.084, 0.029)	0.341	–.058 (–0.087, –0.03)	< 0.001	–0.051 (–0.078, –0.025)	< 0.001
Q3	–0.059 (–0.114, -0.004)	0.035	–0.115 (–0.144, –0.086)	< 0.001	–0.092 (–0.146, –0.038)	0.001
Q4	–0.121 (–0.169, –0.073)	< 0.001	–0.094 (–0.123, –0.066)	< 0.001	–0.102 (–0.127, –0.077)	< 0.001
*P* for trend		< 0.001		<0.001		< 0.001
MetS score trajectory						
Class 1	Ref.	Ref.	Ref.	Ref.	Ref.	Ref.
Class 2	–0.04 (–0.098, 0.018)	0.179	–0.074 (–0.094, –0.054)	< 0.001	–0.069 (–0.093, –0.045)	< 0.001
Class 3	–0.136 (–0.198, –0.074)	< 0.001	–0.111 (–0.134, –0.089)	< 0.001	–0.114 (–0.134, –0.093)	< 0.001
Class 4	–0.155 (–0.257, –0.054)	0.003	–0.114 (–0.146, –0.081)	< 0.001	–0.118 (–0.150, –0.086)	< 0.001
*P* for trend		< 0.001		<0.001		< 0.001

MetS score trajectory was classified into four classes using K-means clustering based on the first and last measured MetS scores. β Coefficient was estimated using linear mixed models. Adjusted covariates include age, sex, residence, educational level, marital status, physical activity, income, alcohol consumption, current smoking, heart-related diseases, stroke. Ref Reference, MetS score metabolic syndrome score; SD, standard deviation; CI, confidence interval.

**TABLE 3:** Longitudinal association between different classes of MetS score and rate of change in memory.

Characteristic	HMACS		CHARLS		Pooled results	
	β (95% CI)	*p*-value	β (95% CI)	*p*-value	β (95% CI)	*p*-value
MetS score, per SD	–0.049 (–0.075, –0.024)	< 0.001	–0.037 (–0.052, –0.022)	< 0.001	–0.040 (–0.054, –0.027)	< 0.001
MetS score, quartiles						
Q1	Ref.	Ref.	Ref.	Ref.	Ref.	Ref.
Q2	–0.025 (–0.094, 0.043)	0.464	–0.038 (–0.062, –0.015)	0.002	–0.037 (–0.059, –0.014)	0.001
Q3	–0.123 (–0.189, –0.058)	< 0.001	–0.190 (–0.215, –0.166)	< 0.001	–0.163 (–0.228, –0.099)	< 0.001
Q4	–0.114 (–0.181, –0.046)	0.001	–0.132 (–0.156, –0.108)	< 0.001	–0.130 (–0.152, –0.108)	< 0.001
*P* for trend		< 0.001		<0.001		< 0.001
Cumulative MetS score, per SD	–0.02 (–0.031, –0.009)	0.001	–0.012 (–0.015, –0.009)	< 0.001	–0.014 (–0.021, –0.007)	< 0.001
Cumulative MetS score, quartiles						
Q1	Ref.	Ref.	Ref.	Ref.	Ref.	Ref.
Q2	–0.016 (–0.086, 0.053)	0.645	–0.083 (–0.114, –0.051)	< 0.001	–0.057 (–0.121, 0.007)	0.083
Q3	–0.029 (–0.097, 0.039)	0.4	–0.148 (–0.18, –0.116)	< 0.001	–0.092 (–0.209, 0.024)	0.120
Q4	–0.100 (–0.160, -0.041)	0.001	–0.118 (–0.149, –0.086)	< 0.001	–0.114 (–0.142, –0.086)	< 0.001
*P* for trend		0.001		< 0.001		<0.001

β Coefficient was estimated using linear mixed models. Adjusted covariates include age, sex, residence, educational level, marital status, physical activity, income, alcohol consumption, current smoking, heart-related diseases, stroke. Ref, Reference; MetS score, metabolic syndrome score; SD, standard deviation; CI, confidence interval; HMACS, Hubei Memory and Aging Cohort Study; CHARLS, China Health and Retirement Longitudinal Study.

**TABLE 4:** Longitudinal association between different classes of MetS score and rate of change in executive function.

Characteristic	HMACS		CHARLS		Pooled results	
	β (95% CI)	*p*-value	β (95% CI)	*p*-value	β (95% CI)	*p*-value
MetS score, per SD	–0.053 (–0.081, –0.025)	< 0.001	–0.008 (–0.02, 0.005)	0.212	–0.029 (–0.073, 0.015)	0.200
MetS score, quartiles						
Q1	Ref.	Ref.	Ref.	Ref.	Ref.	Ref.
Q2	0.024 (–0.049, 0.097)	0.511	–0.001 (–0.013, 0.012)	0.892	–0.000 (–0.012, 0.011)	0.952
Q3	–0.082 (–0.152, –0.013)	0.02	–0.051 (–0.064, –0.038)	< 0.001	–0.052 (–0.066, –0.039)	< 0.001
Q4	–0.114 (–0.187, –0.041)	0.002	–0.049 (–0.062, –0.037)	< 0.001	–0.071 (–0.132, –0.011)	0.021
*P* for trend		< 0.001		<0.001		0.017
Cumulative MetS score, per SD	–0.018 (–0.029, -0.006)	0.002	–0.004 (–0.006, –0.002)	< 0.001	–0.010 (–0.023, 0.004)	0.157
Cumulative MetS score, quartiles						
Q1	Ref.	Ref.	Ref.	Ref.	Ref.	Ref.
Q2	–0.043 (–0.117, 0.032)	0.262	–0.009 (–0.025, 0.008)	0.308	–0.010 (–0.026, 0.005)	0.182
Q3	–0.08 (–0.151, –0.008)	0.028	–0.039 (–0.055, –0.022)	< 0.001	–0.045 (–0.072, –0.017)	0.002
Q4	–0.129 (–0.19, –0.068)	< 0.001	–0.031 (–0.048, –0.015)	< 0.001	–0.075 (–0.171, 0.020)	0.122
*P* for trend		< 0.001		<0.001		0.092

β Coefficient was estimated using linear mixed models. Adjusted covariates include age, sex, residence, educational level, marital status, physical activity, income, alcohol consumption, current smoking, heart-related diseases, and stroke. Ref, Reference; MetS score, metabolic syndrome score; SD, standard deviation; CI, confidence interval; HMACS, Hubei Memory and Aging Cohort Study; CHARLS, China Health and Retirement Longitudinal Study.

### 3.3 Association between cumulative MetS score and cognitive decline

We calculated the Cumulative MetS score to examine the association between cumulative burden of MetS and cognitive decline. As shown in Table 2, in fully adjusted linear mixed models, a 1-SD increase in cumulative MetS score was significantly associated with a faster decline in overall cognitive function in both the HMACS and CHARLS cohorts [*β*_HMACS_ = -0.020 (-0.029, -0.011); *β*_CHARLS_ = -0.010 (-0.013, -0.007), pooled *β* = -0.014 (-0.023, -0.004) z-score/year]. When the Cumulative MetS score was categorized into quartiles, participants in the highest quartile (Q4) showed a significantly faster cognitive decline compared with those in the lowest quartile (Q1) [*β*_HMACS_ = -0.121 (-0.169, -0.073); *β*_CHARLS_ = -0.094 (-0.123, -0.066), pooled *β* = -0.102 (-0.127, -0.077) z-score/year], with a significant linear trend observed (*P*_HMACS_ < 0.001, *P*_CHARLS_ < 0.001, pooled *P* < 0.001). Similarly, as shown in Table 3, for memory function, after full adjustment, a 1-SD increase in Cumulative MetS score was significantly associated with a faster decline in memory performance in both HMACS and CHARLS [*β*_HMACS_ = -0.020 (-0.031, -0.009); *β*_CHARLS_ = -0.012 (-0.015, -0.009), pooled *β* = -0.014 (-0.021, -0.007) z-score/year]. When analyzed by quartiles, participants in Q4 experienced a faster decline in memory function compared with Q1 in both cohorts and in the pooled analysis [*β*_HMACS_ = -0.100 (-0.16, -0.041]; *β*_CHARLS_ = -0.118 (-0.149, -0.086); pooled *β* = -0.114 (-0.142, -0.086) z-score/year], with a significant linear trend (*P*_HMACS_ = 0.001, *P*_CHARLS_ < 0.001, pooled *P* < 0.001). For executive function, as shown in Table 4, a 1-SD increase in Cumulative MetS score was significantly associated with a faster decline in both HMACS and CHARLS after full adjustment [*β*_HMACS_ = -0.018 (-0.029, -0.006); *β*_CHARLS_ = -0.004 (-0.006, -0.002) z-score/year). When categorized into quartiles, participants in Q4 exhibited a faster decline in executive function compared with Q1 [*β*_HMACS_ = -0.129 (-0.19, -0.068); *β*_CHARLS_ = -0.031 (-0.048, -0.015) z-score/year]. However, the pooled results were not statistically significant.

### 3.4 Stratified analyses

**FIGURE 1:**
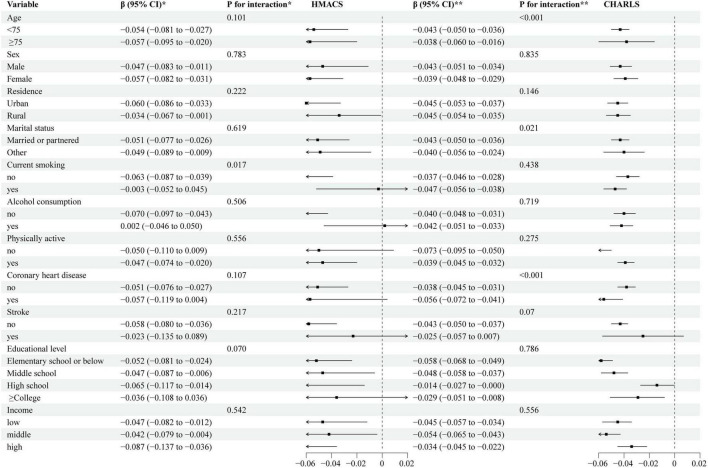
Association of MetS score with global cognitive function trajectory stratified by participant characteristics. β Coefficient was estimated using linear mixed models, with negative values indicating accelerated cognitive decline. Adjusted covariates included age, sex, residence, educational level, marital status, physical activity, income, alcohol consumption, current smoking, heart-related diseases, stroke, *characteristics from the Hubei Memory and Aging Cohort Study, **characteristics from the China Health and Retirement Longitudinal Study.

As shown in Figure 1, stratified analyses revealed a consistent negative association between MetS score and the rate of cognitive decline across most subgroups in both the HMACS and CHARLS cohorts. In the HMACS, smoking status significantly modified the association between MetS score and overall cognitive decline (interaction *P* = 0.017). In the CHARLS cohort, significant interaction effects were observed for age (interaction *P* < 0.001), marital status (interaction *P* = 0.021), and heart-related diseases (interaction *P* < 0.001). Specifically, a 1-SD increase in MetS score was more strongly associated with faster cognitive decline among participants aged < 75 years [β = -0.043 (-0.05, -0.036) z-score/year], married or partnered individuals [β = -0.043 (-0.05, -0.036) z-score/year], and those with heart-related diseases [β = -0.056 (-0.072, -0.041) z-score/year]. In stratified analyses by individual metabolic components, we examined whether these components modified the association between MetS score and cognitive decline by including interaction terms in linear mixed models (Supplementary Table 6). No significant interaction was observed for high blood pressure (interaction *P* = 0.192). The association between MetS score and cognitive decline was similar in participants with and without hypertension. Significant interactions were found for high blood glucose, elevated triglycerides, increased waist circumference, and low HDL-C (all interaction *P* < 0.001). In general, the association between higher MetS score and faster cognitive decline remained significant across most subgroups but the magnitude of association appeared stronger in participants without these abnormalities, elevated triglycerides, increased waist circumference, or low HDL-C.

### 3.5 Sensitivity analyses

**FIGURE 2:**
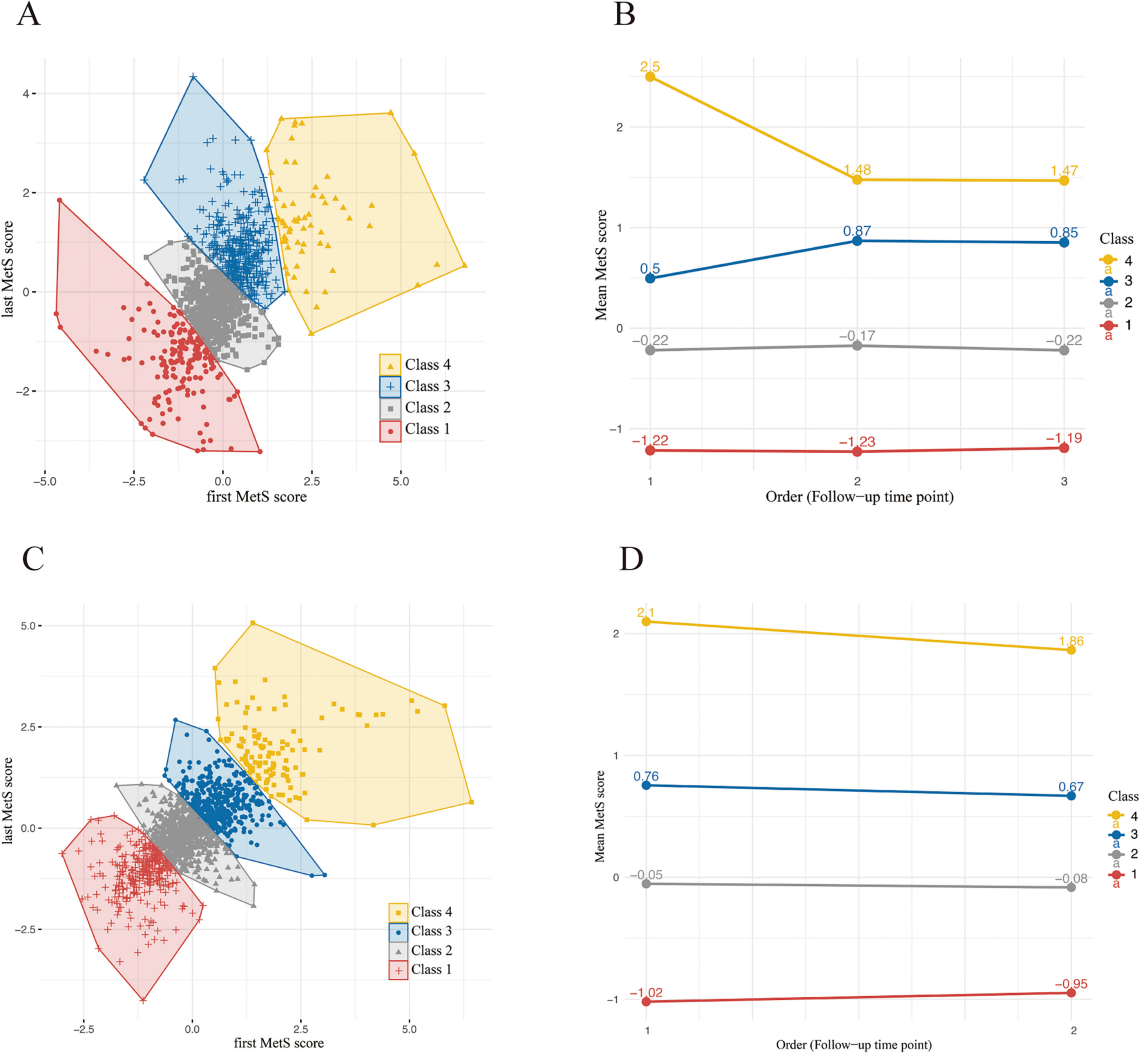
Sensitivity analysis. **(A)** Change in mean MetS score from the first to the third survey across the four k-means clusters in HMACS. **(B)** The longitudinal changes in MetS score between the first and third follow-ups of HMACS were analyzed and visualized using k-means clustering. **(C)** Change in mean MetS score between 2011 and 2015 across the four k-means-derived clusters in CHARLS. **(D)** The changes in MetS score from 2011 to 2015 of CHARLS were visualized by k-means clustering analysis. HMACS Hubei Memory and Aging Cohort Study, CHARLS China Health and Retirement Longitudinal Study.

The sensitivity analyses demonstrated largely robust results. Using the K-means clustering method, MetS score trajectories were reclassified into four groups: Class 1 (lowest MetS score), Class 2 (low MetS score), Class 3 (high MetS score), and Class 4 (highest MetS score) (Figure 2). As shown in Table 2, findings were consistent with the main analyses, in both HMACS and CHARLS; participants in Class 4 showed a faster decline in overall cognitive function compared with those in Class 1. Similar results were obtained when the main analyses were repeated in a restricted sample that excluded participants younger than 65 years and those with cognitive impairment at baseline (Supplementary Table 4).

## 4 Discussion

Our longitudinal study, drawing on two large population cohorts (HMACS and CHARLS), applied a continuous MetS score in place of a binary classification, and revealed a robust and consistent association between higher baseline MetS scores and an accelerated decline in global cognitive function. This association persisted after full adjustment for potential confounders and was further corroborated by extensive sensitivity analyses. Of note, the link was more pronounced for memory function, whereas associations with executive function were less consistent and primarily observed in separate cohort-specific analyses. The finding that MetS appears to preferentially impair memory function early on underscores its potential as a key target for interventions aimed at slowing cognitive decline. From a methodological and study design perspective, our findings provide an important complement to previous studies and offer new evidence to help explain the inconsistent results reported in Chinese populations. These findings support the importance of metabolic health management in older adults, specifically through the control of abdominal obesity, dysglycemia, dyslipidemia, and elevated blood pressure, as a promising approach to mitigate cognitive decline in older adults. Incorporating metabolic health assessments into existing cognitive decline screening strategies, particularly within primary care and community health settings, could thus offer substantial practical value.

Several studies have explored the relationship between MetS and cognitive function, but mostly based on the binary classification of MetS. For example, findings from three large population-based studies conducted in the United Kingdom and South Korea have shown that MetS is not only associated with cognitive decline but may also increase the overall risk of dementia events (Lee et al., 2020; Machado-Fragua et al., 2022; Qureshi et al., 2024a). Each component of MetS has also been linked to a higher risk of developing dementia (Livingston et al., 2020), and the presence of four or five MetS components is significantly associated with dementia (Qureshi et al., 2024a). Furthermore, results from the Study of Women’s Health Across the Nation (SWAN) (Kazlauskaite et al., 2020) and the Singapore Longitudinal Aging Studies (Przybycien-Gaweda et al., 2020) indicated that MetS was significantly associated with greater declines in memory, executive, and global cognitive functions. Despite these findings, the conventional binary diagnostic model of MetS, based solely on its presence or absence, fails to accurately capture the actual severity of metabolic disturbances and overlooks demographic variations. To address this limitation, Yang et al., 2023 developed an age-sex-ethnicity-specific MetS score tailored for the Chinese population. This scoring system accounts for the heterogeneity of metabolic abnormalities and demographic characteristics, providing a reliable tool for assessing and monitoring the severity and progression of MetS in Chinese adults. Our study fills an important gap in existing research by quantifying MetS severity and examining its association with cognitive decline among Chinese adults, offering new insights into the personalized diagnosis and management of MetS.

Our study highlights the significant inverse association between the severity of MetS and cognitive function, particularly in the domain of memory, which is consistent with previous research (Cho et al., 2021; Lee et al., 2020; Tian et al., 2025). The association between increased MetS severity and declines in executive function was observed only in the HMACS and CHARLS cohorts separately, suggesting that future studies with larger sample sizes and longer follow-up periods are needed to confirm these findings. MetS represents a chronic pathological condition characterized by persistent inflammation, hyperinsulinemia, glucose and lipid metabolism disorders, vascular injury, and oxidative stress, with insulin resistance as its core mechanism (Fahed et al., 2022). Chronic peripheral hyperinsulinemia caused by insulin resistance ultimately reduces insulin levels in the brain and induces desensitization of neuronal insulin receptors, which impairs Aβ clearance and increases tau protein hyperphosphorylation. These processes promote neurofibrillary tangle formation and contribute to cognitive impairment (Dao et al., 2023). In addition, chronic inflammation associated with insulin resistance and obesity may reduce metabolic waste clearance, trigger oxidative stress, and disrupt neuroendocrine function, further impairing brain function (Huang S. H. et al., 2022). A recent clinical study in patients with type 2 diabetes reported that reduced volumes in hippocampal subfields are associated with declines in both memory and executive function (Wu et al., 2025). This suggests that the metabolic–cognitive link is not limited to global cognitive scores but may instead reflect damage to memory-related anatomical structures, particularly the hippocampus. The hippocampus is a key region for memory, especially episodic memory, recall, learning and storage processes. It is also highly sensitive to glucose and energy metabolism, insulin signaling, synaptic plasticity, and neural network connectivity. Therefore, metabolic abnormalities are more likely to impair the hippocampus and manifest first and most prominently as memory decline (Minhas et al., 2024). In contrast, executive function depends more heavily on the prefrontal cortex (Menon and D’Esposito, 2022). Taken together, these findings indicate that the impact of MetS on memory function may be more focused and sensitive.

In the stratified analysis of the CHARLS cohort, the association between higher MetS severity and cognitive decline was more pronounced among participants aged < 75 years, married or partnered individuals and those with heart-related diseases. The findings in participants aged ≥ 75 years and in other marital status groups may be explained by the relatively small sample sizes in these subgroups, which may have limited the statistical power. However, previous studies also support a close link between cardiometabolic disorders and cognitive decline. The Swedish National Study on Aging and Care in Kungsholmen (SNAC-K) reported that cardiometabolic diseases were associated with faster cognitive decline and nearly doubled the risk of cognitive impairment and progression to dementia (Dove et al., 2023). In a large systematic review including more than one million participants, individuals with a history of coronary heart disease had a higher risk of dementia compared with those without such a history (relative risk 1.27, 95% CI 1.07–1.50) (Wolters et al., 2018). Accumulating evidence from epidemiological and basic research shows that heart health and brain health are closely linked through both modifiable and non-modifiable factors (American Heart Association scientific statement). Mechanistically, coronary heart disease and vascular risk factors may affect brain health through systemic and neuroinflammation, blood–brain barrier dysfunction, and impaired cerebral microcirculation. These processes may promote amyloid deposition, oxidative stress, and neuroinflammation, leading to reduced cerebral blood flow and vascular dysfunction. As a result, vascular injury and neurodegeneration may interact and worsen each other. In addition, genetic susceptibility and cardiac dysfunction may also contribute to the development and progression of cognitive decline (Iadecola et al., 2023; Iadecola, 2017; Severino et al., 2020). The HMACS results indicated that smoking status may modify the relationship between MetS score and cognitive decline. However, this association was not observed in the CHARLS cohort. Considering the small proportion of smokers in the HMACS sample, the statistical power to detect significant associations may have been limited, or biased estimates may have occurred. Further analyses of cumulative MetS scores and their dynamic changes during follow-up revealed a dose–response relationship between cumulative MetS score and cognitive decline, particularly in memory performance. Classification based on K-means clustering in both HMACS and CHARLS showed that participants with the highest MetS scores experienced the fastest cognitive decline. Despite some variations, the overall pattern was consistent with the baseline level of metabolic dysregulation. Although the pooled analysis did not reach statistical significance, future research should further explore sources of heterogeneity across cohorts to enhance the ability to capture true effects.

In stratified analyses by metabolic components, we observed that the association between MetS score and cognitive decline was stronger among participants without certain adverse metabolic components. Several explanations may account for this finding. First, a potential ceiling effect may exist. Individuals with established metabolic abnormalities may already be at elevated risk of cognitive decline, such that additional increases in the MetS score confer relatively smaller incremental effects. In contrast, among participants without these abnormalities, variation in the MetS score may better capture the cumulative metabolic burden, resulting in a more pronounced association with cognitive trajectories. Second, individuals with diagnosed metabolic disorders are more likely to receive medical treatment or lifestyle interventions, which may attenuate the detrimental impact of metabolic burden on cognitive function. Third, unequal subgroup sample sizes may have influenced the observed differences. Smaller subgroups (e.g., participants with elevated triglycerides) may have had reduced statistical power and less precise effect estimates, potentially contributing to apparent heterogeneity across strata. Finally, residual confounding and differences in baseline risk profiles between subgroups cannot be completely excluded. Therefore, these findings should be interpreted cautiously and warrant further investigation.

Our study presents several notable strengths. First, our data were derived from two large-scale, prospective cohort studies. Second, we applied a newly developed age-, sex-, and ethnicity-specific MetS severity score to quantify the degree of MetS, making it more suitable for the Chinese population. This approach accounts for demographic influences such as age, sex, and ethnicity, and offers advantages including non-invasiveness, accessibility, and cost-effectiveness. To our knowledge, this is the first study to link a Chinese-specific quantitative measure of MetS severity with cognitive function. Moreover, we calculated the cumulative MetS score, providing deeper insight into the relationship between the sustained severity of MetS and cognitive decline. However, several limitations should be noted. Due to the constraints of the CHARLS database, only two complete sets of metabolic data were available, and the relatively short follow-up period may have limited our ability to fully capture the impact of changes in MetS score on cognitive decline. Additionally, differences between the two cohorts in survey timing, follow-up duration, cognitive assessments, and methods for collecting metabolic indicators may have influenced effect estimates. The HMACS cohort has a regional focus, with participants primarily drawn from community-dwelling older adults in a specific area of Hubei Province, which may limit the generalizability of its findings. Although CHARLS is a nationally representative cohort with wide coverage, issues such as loss to follow-up and missing data among some participants may have affected the robustness of the results. Furthermore, since our study included only Chinese participants and used a population-specific formula, the findings may not be generalizable to other countries or ethnic groups. We also cannot rule out the influence of unmeasured or residual confounding factors. Chronic conditions (e.g., diabetes and persistent inflammation), emotional status, and other lifestyle factors may affect both baseline MetS severity and cognitive decline. Finally, as this was an observational study, causal relationships cannot be established, and the potential mediating mechanisms between exposure and outcome remain to be fully elucidated, warranting further investigation. Future studies with longer follow-up durations are needed to better capture long-term cognitive trajectories. Moreover, integrating multi-omics data and incorporating neurodegenerative biomarkers, such as cerebrospinal fluid Aβ and tau proteins, may help clarify the molecular mechanisms underlying the association between metabolic burden and cognitive decline.

## 5 Conclusion

Higher baseline and cumulative MetS scores were associated with faster declines in cognitive function among adults aged 60 years and older, particularly in memory function. These findings underscore the potential value of monitoring MetS scores to identify high-risk individuals early and to guide prevention and management strategies.

## Data Availability

The raw data supporting the conclusions of this article will be made available by the authors, without undue reservation.

## Footnotes

1
www.chictr.org.cn
