# Supplementary material for: Association of metabolic syndrome severity with cognitive decline among Chinese older adults: evidence from two prospective cohort studies
Source: Front Neurosci. 2026 Mar 17;20:1780804. doi: 10.3389/fnins.2026.1780804 (PMC13036125; doi:10.3389/fnins.2026.1780804)
Supplement: Supplementary file 1 [file Data_sheet_1.pdf]

Supplementary Material

1.1 Supplementary Figures

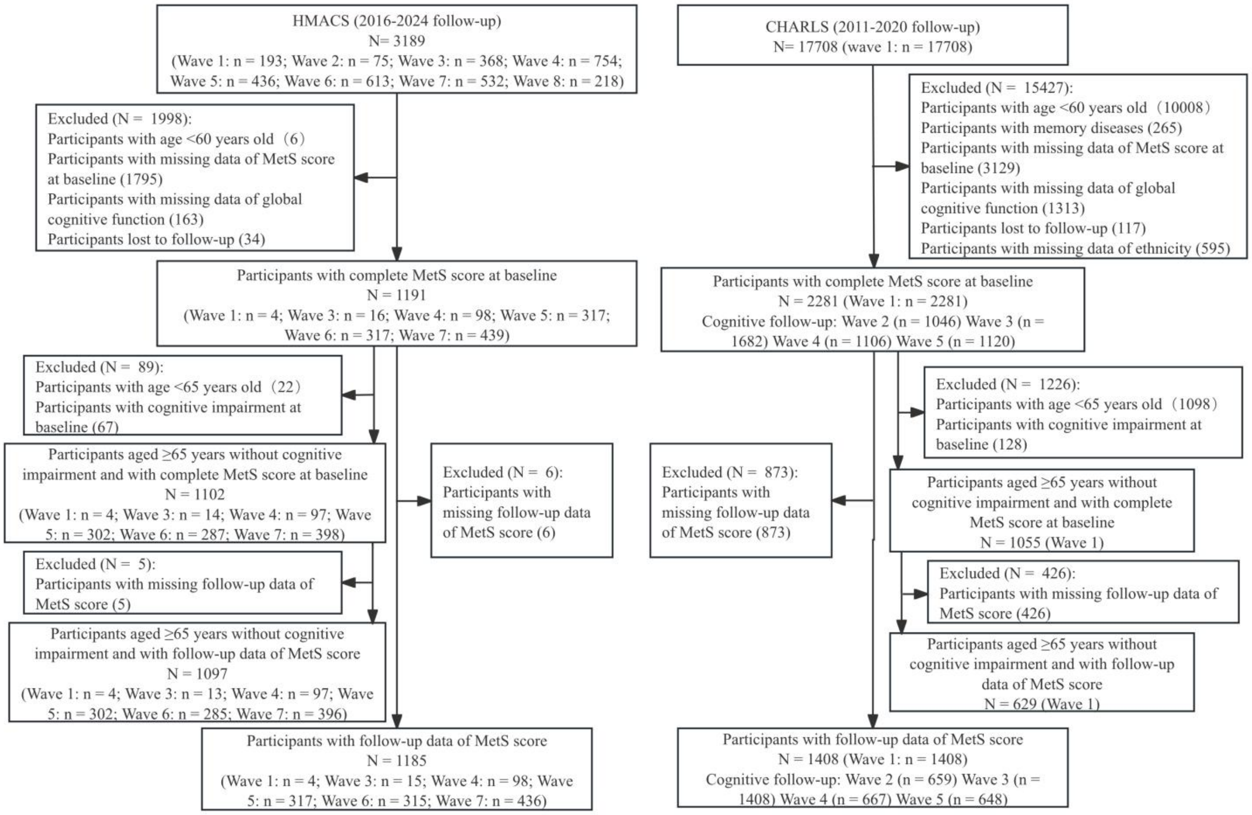

Supplementary Figure 1. Flowchart of the population included in our final analysis.

Supplementary Table 1 Baseline characteristics of the study population by quartiles of cumulative MetS score in HMACS (n = 1185) and CHARLS (n = 1408).

**Characteristic**	**Quartiles of cumulative MetS score**					***p-value***
	**Overall**	**Q1**	**Q2**	**Q3**	**Q4**	
HMACS						
N	1185	297	296	296	296	
Age, mean (SD), year	72.0 (5.2)	72.2 (5.3)	72.2 (5.4)	72.2 (5.0)	71.1 (5.0)	0.017
Age, n (%)						0.001
<75	838 (70.7)	199 (67.0)	196 (66.2)	206 (69.6)	237 (80.1)	
≥75	347 (29.3)	98 (33.0)	100 (33.8)	90 (30.4)	59 (19.9)	
Female, n (%)	698 (58.9)	183 (61.6)	169 (57.1)	176 (59.5)	170 (57.4)	0.658
Residence, n (%)						<0.001
Urban	701 (59.2)	194 (65.3)	140 (47.3)	177 (59.8)	190 (64.2)	
Rural	484 (40.8)	103 (34.7)	156 (52.7)	119 (40.2)	106 (35.8)	
Educational level, n (%)						0.101
Elementary school or below	503 (42.4)	118 (39.7)	150 (50.7)	120 (40.5)	115 (38.9)	
Middle school	269 (22.7)	73 (24.6)	54 (18.2)	71 (24.0)	71 (24.0)	
High school	259 (21.9)	63 (21.2)	65 (22.0)	64 (21.6)	67 (22.6)	
≥College	154 (13.0)	43 (14.5)	27 (9.1)	41 (13.9)	43 (14.5)	
Marital status, n (%)						0.430
Married or partnered	862 (73.6)	205 (70.0)	219 (75.0)	216 (74.2)	222 (75.3)	
Other	309 (26.4)	88 (30.0)	73 (25.0)	75 (25.8)	73 (24.7)	
Physically active, n (%)	848 (72.2)	217 (73.8)	202 (68.9)	214 (73.0)	215 (73.1)	0.540
Alcohol consumption, n (%)	322 (27.5)	83 (28.4)	87 (29.7)	72 (24.7)	80 (27.2)	0.567
Current smoking, n (%)	303 (25.8)	77 (26.2)	85 (29.0)	60 (20.4)	81 (27.6)	0.088
Income, n (%)						0.004

Supplementary Table 1 (continued).

**Characteristic**	**Quartiles of cumulative MetS score**					***p-value***
	**Overall**	**Q1**	**Q2**	**Q3**	**Q4**	
Low	394 (34.9)	92 (32.1)	123 (44.6)	87 (31.1)	92 (32.1)	
Middle	453 (40.1)	129 (44.9)	95 (34.4)	119 (42.5)	110 (38.3)	
High	283 (25.0)	66 (23.0)	58 (21.0)	74 (26.4)	85 (29.6)	
Heart-related diseases, n (%)	227 (19.5)	48 (16.4)	61 (21.0)	58 (19.9)	60 (20.8)	0.464
Stroke, n (%)	212 (18.3)	39 (13.4)	56 (19.2)	61 (21.0)	56 (19.5)	0.089
Han ethnicity, n(%)	1185 (100)	297 (100)	296 (100)	296 (100)	296 (100)	
MetS score, mean (SD)	0.196 (0.864)	-0.585 (0.651)	-0.103 (0.343)	0.427 (0.424)	1.048 (0.887)	
CHARLS						
N	1408	331	353	356	368	
Age, mean (SD), year	65.6 (4.8)	66.0 (5.0)	65.8 (5.1)	65.6 (4.7)	65.2 (4.6)	0.204
Age, n (%)						0.513
<75	1325 (94.1)	307 (92.7)	331 (93.8)	336 (94.4)	351 (95.4)	
≥75	83 (5.9)	24 (7.3)	22 (6.2)	20 (5.6)	17 (4.6)	
Female, n (%)	651 (46.2)	112 (33.8)	168 (47.6)	172 (48.3)	199 (54.1)	<0.001
Residence, n (%)						<0.001
Urban	896 (63.6)	243 (73.4)	246 (69.7)	210 (59.0)	197 (53.5)	
Rural	512 (36.4)	88 (26.6)	107 (30.3)	146 (41.0)	171 (46.5)	
Educational level, n (%)						0.620
Elementary school or below	653 (46.4)	153 (46.2)	166 (47.0)	177 (49.7)	157 (42.7)	
Middle school	441 (31.3)	108 (32.6)	108 (30.6)	101 (28.4)	124 (33.7)	
High school	226 (16.1)	50 (15.1)	62 (17.6)	52 (14.6)	62 (16.8)	
≥College	88 (6.2)	20 (6.0)	17 (4.8)	26 (7.3)	25 (6.8)	
Marital status, n (%)						0.744
Married or partnered	1214 (86.2)	290 (87.6)	299 (84.7)	307 (86.2)	318 (86.4)	
Other	194 (13.8)	41 (12.4)	54 (15.3)	49 (13.8)	50 (13.6)	
Physically active, n (%)	1229 (87.4)	299 (90.3)	305 (86.4)	309 (87.0)	316 (86.1)	0.320

Supplementary Table 1 (continued).

**Characteristic**	**Quartiles of cumulative MetS score**					***p-value***
	**Overall**	**Q1**	**Q2**	**Q3**	**Q4**	
Alcohol consumption, n (%)	625 (44.4)	178 (53.8)	149 (42.2)	151 (42.5)	147 (39.9)	0.001
Current smoking, n (%)	630 (44.7)	187 (56.5)	144 (40.8)	143 (40.2)	156 (42.4)	<0.001
Income, n (%)						0.045
Low	409 (31.8)	111 (37.2)	108 (32.7)	90 (28.0)	100 (29.9)	
Middle	459 (35.7)	102 (34.2)	129 (39.1)	111 (34.5)	117 (34.9)	
High	417 (32.5)	85 (28.5)	93 (28.2)	121 (37.6)	118 (35.2)	
Heart-related diseases, n (%)	214 (15.3)	35 (10.6)	52 (14.9)	56 (15.8)	71 (19.3)	0.016
Stroke, n (%)	39 (2.8)	8 (2.4)	9 (2.6)	8 (2.2)	14 (3.8)	0.565
Han ethnicity, n(%)	1325 (94.1)	305 (92.1)	336 (95.2)	337 (94.7)	347 (94.3)	0.354
MetS score, mean (SD)	0.447 (0.985)	-0.653 (0.478)	0.134 (0.346)	0.591 (0.337)	1.598 (0.869)	

Categorical variables were presented as numbers (percentage, %), and continuous variables were presented as mean (standard deviation, SD). P values for differences between groups were derived using Pearson's Chi-squared test for categorical variables and the Kruskal–Wallis rank sum test for continuous variables. This other marital status refers to divorced, separated, widowed, or never married statuses

MetS score, metabolic syndrome score. *SD,* standard deviation. HMACS, Hubei Memory and Aging Cohort Study, CHARLS, China Health and Retirement Longitudinal Study.

Supplementary Table 2 Baseline characteristics of the study population by quartiles of baseline MetS score (excluding participants younger than 65 years and those with cognitive impairment) in the HMACS (N = 1102) and the CHARLS (N = 1055).

**Characteristic**	**Quartiles of baseline MetS score**					***p-value***
	**Overall**	**Q1**	**Q2**	**Q3**	**Q4**	
HMACS						
N	1102	276	276	275	275	
Age, mean (SD), year	72.0 (5.1)	72.5 (5.3)	72.2 (5.1)	71.7 (5.0)	71.5 (5.0)	0.100
Age, n (%)						0.066
<75	781 (70.9)	186 (67.4)	186 (67.4)	200 (72.7)	209 (76.0)	
≥75	321 (29.1)	90 (32.6)	90 (32.6)	75 (27.3)	66 (24.0)	
Female, n (%)	639 (58.0)	163 (59.1)	167 (60.5)	152 (55.3)	157 (57.1)	0.621
Residence, n (%)						<0.001
Urban	680 (61.7)	132 (47.8)	167 (60.5)	193 (70.2)	188 (68.4)	
Rural	422 (38.3)	144 (52.2)	109 (39.5)	82 (29.8)	87 (31.6)	
Educational level, n (%)						0.002
Elementary school or below	438 (39.7)	139 (50.4)	114 (41.3)	87 (31.6)	98 (35.6)	
Middle school	262 (23.8)	57 (20.7)	66 (23.9)	75 (27.3)	64 (23.3)	
High school	252 (22.9)	51 (18.5)	54 (19.6)	75 (27.3)	72 (26.2)	
≥College	150 (13.6)	29 (10.5)	42 (15.2)	38 (13.8)	41 (14.9)	
Marital status, n (%)						<0.001
Married or partnered	808 (74.1)	175 (64.1)	216 (79.1)	211 (77.0)	206 (76.0)	
Other	283 (25.9)	98 (35.9)	57 (20.9)	63 (23.0)	65 (24.0)	
Physically active, n (%)	809 (74.2)	184 (67.2)	203 (74.6)	214 (78.1)	208 (76.8)	0.017
Alcohol consumption, n (%)	304 (27.9)	83 (30.4)	76 (28.0)	71 (26.0)	74 (27.3)	0.709
Current smoking, n (%)	288 (26.4)	78 (28.5)	68 (25.0)	73 (26.6)	69 (25.4)	0.793
Income, n (%)						<0.001
Low	396 (37.6%)	132 (50.4%)	104 (40.2%)	75 (28.1%)	85 (32.1%)	
Middle	376 (35.7%)	80 (30.5%)	91 (35.1%)	105 (39.3%)	100 (37.7%)	
High	281 (26.7%)	50 (19.1%)	64 (24.7%)	87 (32.6%)	80 (30.2%)	

Supplementary Table 2 (continued).

**Characteristic**	**Quartiles of baseline MetS score**					***p-value***
	**Overall**	**Q1**	**Q2**	**Q3**	**Q4**	
Heart-related diseases, n (%)	209 (19.3)	52 (19.0)	51 (19.0)	53 (19.3)	53 (19.9)	0.992
Stroke, n (%)	194 (18.0)	40 (14.7)	57 (21.3)	52 (19.0)	45 (17.0)	0.228
Han ethnicity, n(%)	1102 (100)	276 (100)	276 (100)	275 (100)	275 (100)	
MetS score, mean (SD)	0.280 (0.860)	-0.506 (0.747)	0.031 (0.365)	0.400 (0.275)	1.200 (0.809)	
CHARLS						
N	1055	261	272	264	258	
Age, mean (SD), year	69.6 (4.0)	69.6 (3.7)	69.3 (3.6)	70.1 (4.7)	69.4 (4.0)	0.104
Age, n (%)						0.149
<75	923 (87.5)	228 (87.4)	248 (91.2)	224 (84.8)	223 (86.4)	
≥75	132 (12.5)	33 (12.6)	24 (8.8)	40 (15.2)	35 (13.6)	
Female, n (%)	423 (40.1)	81 (31.0)	95 (34.9)	105 (39.8)	142 (55.0)	<0.001
Residence, n (%)						<0.001
Urban	634 (60.1)	168 (64.4)	183 (67.3)	156 (59.1)	127 (49.2)	
Rural	421 (39.9)	93 (35.6)	89 (32.7)	108 (40.9)	131 (50.8)	
Educational level, n (%)						0.652
Elementary school or below	453 (42.9)	112 (42.9)	112 (41.2)	113 (42.8)	116 (45.0)	
Middle school	348 (33.0)	89 (34.1)	101 (37.1)	81 (30.7)	77 (29.8)	
High school	161 (15.3)	38 (14.6)	38 (14.0)	48 (18.2)	37 (14.3)	
≥College	93 (8.8)	22 (8.4)	21 (7.7)	22 (8.3)	28 (10.9)	
Marital status, n (%)						0.194
Married or partnered	859 (81.4)	221 (84.7)	225 (82.7)	205 (77.7)	208 (80.6)	
Other	196 (18.6)	40 (15.3)	47 (17.3)	59 (22.3)	50 (19.4)	
Physically active, n (%)	906 (86.0)	233 (89.3)	237 (87.1)	223 (84.8)	213 (82.9)	0.170
Alcohol consumption, n (%)	498 (47.2)	151 (57.9)	121 (44.5)	124 (47.0)	102 (39.5)	<0.001
Current smoking, n (%)	503 (47.7)	135 (51.7)	134 (49.3)	133 (50.4)	101 (39.1)	0.016
Income, n (%)						0.246

Supplementary Table 2 (continued)

**Characteristic**	**Quartiles of baseline MetS score**					***p-value***
	**Overall**	**Q1**	**Q2**	**Q3**	**Q4**	
Low	316 (32.2)	80 (33.2)	91 (36.3)	82 (32.8)	63 (26.4)	
Middle	345 (35.2)	91 (37.8)	82 (32.7)	86 (34.4)	86 (36.0)	
High	320 (32.6)	70 (29.0)	78 (31.1)	82 (32.8)	90 (37.7)	
Heart-related diseases, n (%)	159 (15.1)	31 (11.9)	36 (13.3)	35 (13.4)	57 (22.1)	0.004
Stroke, n (%)	31 (2.9)	6 (2.3)	3 (1.1)	11 (4.2)	11 (4.3)	0.087
Han ethnicity, n(%)	1005 (95.3)	248 (95.0)	261 (96.0)	254 (96.2)	242 (93.8)	0.557
MetS score, mean (SD)	0.300 (1.041)	-0.833 (0.395)	-0.041 (0.180)	0.487 (0.161)	1.614 (1.014)	

Categorical variables were presented as numbers (percentage, %), and continuous variables were presented as means (standard deviation, SD). P values for differences between groups were derived using Pearson's Chi-squared test for categorical variables and the Kruskal–Wallis rank sum test for continuous variables. This other marital status refers to divorced, separated, widowed, or never married statuses.

MetS score, metabolic syndrome score. *SD* standard deviation. HMACS, Hubei Memory and Aging Cohort Study, CHARLS, China Health and Retirement Longitudinal Study.

Supplementary Table 3 Baseline characteristics of the study population by quartiles of cumulative MetS score (excluding participants younger than 65 years and those with cognitive impairment) in HMACS (N = 1097) and CHARLS (N = 629).

**Characteristic**	**Quartiles of cumulative MetS score**					***p-value***
	**Overall**	**Q1**	**Q2**	**Q3**	**Q4**	
HMACS						
N	1097	275	274	274	274	
Age, mean (SD), year	72.0 (5.1)	72.2 (5.4)	72.3 (5.3)	72.0 (4.7)	71.3 (4.9)	0.084
Age, n (%)						<0.001
<75	780 (71.1)	188 (68.4)	176 (64.2)	196 (71.5)	220 (80.3)	
≥75	317 (28.9)	87 (31.6)	98 (35.8)	78 (28.5)	54 (19.7)	
Female, n (%)	636 (58.0)	168 (61.1)	163 (59.5)	155 (56.6)	150 (54.7)	0.432
Residence, n (%)						<0.001
Urban	680 (62.0)	173 (62.9)	141 (51.5)	171 (62.4)	195 (71.2)	
Rural	417 (38.0)	102 (37.1)	133 (48.5)	103 (37.6)	79 (28.8)	
Educational level, n (%)						0.206
Elementary school or below	434 (39.6)	112 (40.7)	126 (46.0)	106 (38.7)	90 (32.8)	
Middle school	262 (23.9)	67 (24.4)	58 (21.2)	68 (24.8)	69 (25.2)	
High school	251 (22.9)	56 (20.4)	60 (21.9)	64 (23.4)	71 (25.9)	
≥College	150 (13.7)	40 (14.5)	30 (10.9)	36 (13.1)	44 (16.1)	
Marital status, n (%)						0.045
Married or partnered	806 (74.2)	188 (69.4)	196 (71.8)	212 (78.5)	210 (77.2)	
Other	280 (25.8)	83 (30.6)	77 (28.2)	58 (21.5)	62 (22.8)	
Physically active, n (%)	804 (74.0)	197 (72.4)	195 (71.7)	202 (74.5)	210 (77.5)	0.413
Alcohol consumption, n (%)	301 (27.8)	81 (30.0)	76 (27.9)	69 (25.7)	75 (27.6)	0.734
Current smoking, n (%)	286 (26.3)	73 (26.8)	68 (25.0)	72 (26.6)	73 (26.8)	0.955
Income, n (%)						0.002
Low	392 (37.4)	101 (38.3)	120 (46.3)	94 (36.2)	77 (29.1)	
Middle	376 (35.9)	102 (38.6)	75 (29.0)	98 (37.7)	101 (38.1)	
High	280 (26.7)	61 (23.1)	64 (24.7)	68 (26.2)	87 (32.8)	

Supplementary Table 3 (continued)

**Characteristic**	**Quartiles of cumulative MetS score**					***p-value***
	**Overall**	**Q1**	**Q2**	**Q3**	**Q4**	
Heart-related diseases, n (%)	209 (19.4)	51 (18.8)	48 (17.8)	63 (23.2)	47 (17.7)	0.314
Stroke, n (%)	192 (17.9)	39 (14.5)	48 (17.8)	59 (21.9)	46 (17.3)	0.160
Han ethnicity, n(%)	1097 (100)	275 (100)	274 (100)	274 (100)	274 (100)	
MetS score, mean (SD)	0.278 (0.861)	-0.491 (0.691)	0.087 (0.491)	0.437 (0.438)	1.079 (0.873)	
CHARLS						
N	629	152	157	161	159	
Age, mean (SD), year	69.3 (4.0)	69.4 (3.5)	69.2 (4.6)	69.5 (3.8)	69.0 (3.8)	0.662
Age, n (%)						0.892
<75	560 (89.0)	134 (88.2)	140 (89.2)	142 (88.2)	144 (90.6)	
≥75	69 (11.0)	18 (11.8)	17 (10.8)	19 (11.8)	15 (9.4)	
Female, n (%)	372 (59.1)	109 (71.7)	97 (61.8)	93 (57.8)	73 (45.9)	<0.001
Residence, n (%)						0.006
Urban	246 (39.1)	51 (33.6)	49 (31.2)	70 (43.5)	76 (47.8)	
Rural	383 (60.9)	101 (66.4)	108 (68.8)	91 (56.5)	83 (52.2)	
Educational level, n (%)						0.823
Elementary school or below	265 (42.1)	59 (38.8)	68 (43.3)	74 (46.0)	64 (40.3)	
Middle school	209 (33.2)	55 (36.2)	49 (31.2)	49 (30.4)	56 (35.2)	
High school	97 (15.4)	25 (16.4)	28 (17.8)	20 (12.4)	24 (15.1)	
≥College	58 (9.2)	13 (8.6)	12 (7.6)	18 (11.2)	15 (9.4)	
Marital status, n (%)						0.422
Married or partnered	509 (80.9)	130 (85.5)	125 (79.6)	127 (78.9)	127 (79.9)	
Other	120 (19.1)	22 (14.5)	32 (20.4)	34 (21.1)	32 (20.1)	
Physically active, n (%)	542 (86.4)	136 (89.5)	135 (86.0)	135 (84.4)	136 (86.1)	0.610
Alcohol consumption, n (%)	303 (48.2)	90 (59.2)	76 (48.4)	67 (41.6)	70 (44.0)	0.010
Current smoking, n (%)	301 (47.9)	89 (58.6)	76 (48.4)	70 (43.5)	66 (41.5)	0.013
Income, n (%)						0.110
Low	189 (32.0)	57 (40.1)	49 (32.7)	41 (27.0)	42 (28.8)	

Supplementary Table 3 (continued)

**Characteristic**	**Quartiles of cumulative MetS score**					***p-value***
	**Overall**	**Q1**	**Q2**	**Q3**	**Q4**	
Middle	213 (36.1)	49 (34.5)	59 (39.3)	53 (34.9)	52 (35.6)	
High	188 (31.9)	36 (25.4)	42 (28.0)	58 (38.2)	52 (35.6)	
Heart-related diseases, n (%)	101 (16.1)	16 (10.6)	22 (14.1)	29 (18.0)	34 (21.5)	0.053
Stroke, n (%)	20 (3.2)	4 (2.6)	3 (1.9)	4 (2.5)	9 (5.7)	0.278
Han ethnicity, n(%)	597 (94.9)	143 (94.1)	149 (94.9)	157 (97.5)	148 (93.1)	0.310
MetS score, mean (SD)	0.430 (1.050)	-0.686 (0.492)	0.099 (0.292)	0.561 (0.347)	1.690 (1.011)	

Categorical variables were presented as numbers (percentage, %), and continuous variables were presented as means (standard deviation, SD). P values for differences between groups were derived using Pearson's Chi-squared test for categorical variables and the Kruskal–Wallis rank sum test for continuous variables. This other marital status refers to divorced, separated, widowed, or never married statuses.

MetS score metabolic syndrome score. *SD* standard deviation. HMACS Hubei Memory and Aging Cohort Study, CHARLS China Health and Retirement Longitudinal Study.

Supplementary Table 4 Longitudinal association between different classes of MetS score and rate of change in global cognitive function among individuals aged ≥65 years without cognitive impairment.

Characteristic	HMACS		CHARLS		Pooled results	
	β (95% CI)	*p*-value	β (95% CI)	*p*-value	β (95% CI)	*p*-value
MetS score, per SD	-0.043 (-0.065, -0.022)	<0.001	-0.038 (-0.06, -0.015)	0.001	-0.041 (-0.056, -0.025)	<0.001
MetS score, quartiles						
Q1	Ref.	Ref.	Ref.	Ref.	Ref.	Ref.
Q2	0.008 (-0.05, 0.066)	0.778	-0.042 (-0.071, -0.013)	0.004	-0.024 (-0.071, 0.023)	0.324
Q3	-0.062 (-0.118, -0.006)	0.029	-0.188 (-0.218, -0.158)	<0.001	-0.127 (-0.251, -0.004)	0.043
Q4	-0.093 (-0.15, -0.035)	0.002	-0.172 (-0.201, -0.143)	<0.001	-0.136 (-0.213, -0.059)	0.001
P for trend		<0.001		<0.001		<0.001
Cumulative MetS score, per SD	-0.018 (-0.027, -0.008)	<0.001	-0.013 (-0.017, -0.009)	<0.001	-0.014 (-0.017, -0.010)	<0.001
Cumulative MetS score, quartiles						
Q1	Ref.	Ref.	Ref.	Ref.	Ref.	Ref.
Q2	-0.013 (-0.072, 0.046)	0.67	-0.091 (-0.13, -0.052)	<0.001	-0.055 (-0.131, 0.021)	0.156
Q3	-0.07 (-0.127, -0.013)	0.016	-0.173 (-0.213, -0.134)	<0.001	-0.124 (-0.224, -0.023	0.016
Q4	-0.114 (-0.164, -0.065)	<0.001	-0.14 (-0.179, -0.101)	<0.001	-0.130 (-0.160, -0.099)	<0.001
P for trend		<0.001		<0.001		<0.001

β Coefficient was estimated using linear mixed models. Adjusted covariates include age, sex, residence, educational level, marital status, physical activity, income, alcohol consumption, current smoking, heart-related diseases, and stroke

Ref, Reference, *MetS score,* metabolic syndrome score, *SD,* standard deviation, *CI,* confidence interval. HMACS, Hubei Memory and Aging Cohort Study, CHARLS, China Health and Retirement Longitudinal Study.

Supplementary Table 5 Age-sex-ethnicity-specific metabolic syndrome score in the Chinese adults.

**Groups**	**Equations**
Han	
Male	−2.3741 + 0.0264*WC+ 0.4933*TG-0.999*HDL-C+ 0.0054*MAP + 0.0821*FBG
Female	−0.5682 + 0.0153*WC+ 0.4587*TG-1.3567*HDL-C+ 0.0036*MAP+ 0.0688*FBG
Minority	
Male	−3.7784 + 0.0399*WC+ 0.3234*TG-1.0218*HDL-C+ 0.0116*MAP + 0.0738*FBG
Female	−1.5436 + 0.0212*WC+ 0.4698*TG-1.3471*HDL-C+ 0.0084*MAP + 0.0821*FBG

FBG fasting blood glucose (mmol/L), HDL-C high-density lipoprotein cholesterol (mmol/L), MAP mean arterial pressure (mmHg), TG triglycerides (mmol/L), WC waist circumference (cm).

Supplementary Table 6 Stratified analyses of the association between MetS score and the rate of cognitive decline according to individual metabolic components in CHARLS.

Characteristic	MetS score	*p* interaction
	β (95% CI) *p*-value	
High blood pressure		0.192
Yes (n= 1218)	-0.046 (-0.057, -0.036) <0.001	
No (n= 1063)	-0.038 (-0.05, -0.027) <0.001	
High blood glucose		<0.001
Yes (n= 1340)	-0.039 (-0.048, -0.029) <0.001	
No (n= 941)	-0.073 (-0.088, -0.058) <0.001	
Elevated triglycerides		<0.001
Yes (n= 565)	-0.011 (-0.028, 0.005) <0.001	
No (n=1716)	-0.079 (-0.091, -0.066) <0.001	
Increased waist circumference		<0.001
Yes (n= 968)	-0.021 (-0.035, -0.008) 0.002	
No (n= 1313)	-0.052 (-0.063, -0.04) <0.001	
Low HDL-C		<0.001
Yes (n=554)	0.004 (-0.014, 0.022) 0.666	
No (n=1727)	-0.068 (-0.079, -0.056) <0.001	

β Coefficient was estimated using linear mixed models, with negative values indicating accelerated cognitive decline. Adjusted covariates included age, sex, residence, educational level, marital status, physical activity, income, alcohol consumption, current smoking, heart-related diseases, stroke HDL-C high-density lipoprotein cholesterol, CHARLS China Health and Retirement Longitudinal Study.
